# Duplicated Right Testicular Artery: A Cadaveric Case Report and Review of the Literature

**DOI:** 10.7759/cureus.97803

**Published:** 2025-11-25

**Authors:** Katia Costillo, Tyson Dooley, Anna Grote, Holly Jarzynka, Andrew Piron, Uzochukwu Adabanya, Matthew D Overturf

**Affiliations:** 1 Medical School, Edward Via College of Osteopathic Medicine, Monroe, USA; 2 Anatomical Sciences, Edward Via College of Osteopathic Medicine, Monroe, USA

**Keywords:** anatomical variation, arterial duplication, testicular artery, urology, vascular surgery

## Abstract

The testicular artery usually arises as a single branch from the abdominal aorta. Anatomical variations, including duplication, are rare but clinically relevant in urological, vascular, and radiological practices. During routine cadaveric dissections, two male specimens were found to have duplicated right testicular arteries. Each specimen exhibited two distinct arterial branches originating directly from the abdominal aorta, descending independently within the spermatic cord. No associated renal or ureteral anomalies were identified. Duplication of the testicular artery has been documented in anatomical studies and, when present, is most commonly observed on the right side. Embryologically, this anomaly is attributed to the persistence of one or more mesonephric arteries that normally regress during development. Although often asymptomatic, recognition of this variation is essential in orchiectomy, varicocelectomy, renal transplantation, and interventional radiology to prevent inadvertent ischemic injury. This case report highlights duplicated right testicular arteries observed in two different cadavers, emphasizing the importance of awareness of vascular variations in surgical and radiological practice.

## Introduction

The testicular artery (TA), also referred to as the internal spermatic artery, is a paired vessel that typically arises as a single branch from the ventral aspect of the abdominal aorta, just inferior to the origin of the renal arteries. It courses through the retroperitoneal space, enters the inguinal canal via the deep inguinal ring, and exits through the superficial inguinal ring to reach the testis, where it serves as the principal arterial supply responsible for sustaining spermatogenesis and testicular endocrine function.

While the TA most often arises as a solitary trunk, numerous anatomical variations have been reported in the literature regarding its origin, number, and course. These include a high origin from the abdominal aorta, origin from the renal artery, duplication or multiple arteries supplying a single testis, and aberrant trajectories either anterior or posterior to major retroperitoneal vessels. Among these, duplication of the TA is rare and is often discovered incidentally during cadaveric dissection, radiological examination, or retroperitoneal surgery [1-3].

The persistence or regression of mesonephric arteries can elucidate the embryological foundation for variations in the TA. Throughout development, the mesonephros is supplied by several lateral mesonephric branches originating from the dorsal aorta. Usually, only a single artery remains to constitute the definitive gonadal artery, whereas the others regress. Failure in this regression process may lead to duplication or the presence of multiple gonadal arteries [4].

Epidemiologically, the incidence of duplicated TAs is reported to be 2.5-3%, with right-sided duplications more common than left-sided [1]. Bilateral duplications are rare, with only a few cases documented in the literature [3]. Although these variations are often asymptomatic, they hold significant importance in surgical and interventional practices.

Clinically, unrecognized duplication may influence urological procedures such as orchiectomy, varicocelectomy, and testicular tumor resection. Unintentional ligation of an accessory TA may result in testicular ischemia or impaired spermatogenesis. Similarly, during renal transplantation and retroperitoneal surgeries, the presence of multiple gonadal arteries can complicate hilar dissection and vascular anastomosis. From a radiological perspective, duplication may be mistaken for aberrant vasculature on angiography or CT scans, potentially leading to incomplete embolization during procedures such as varicocele treatment [5,6].

This report delineates instances of duplicated right testicular arteries identified in two cadavers and offers a review of pertinent literature. By highlighting these anatomical findings, our objective is to emphasize the significance of understanding gonadal vascular variations within the contexts of clinical anatomy, surgical procedures, and radiology. This is a cadaveric case report and does not require IRB approval.

## Case presentation

Two male cadavers preserved in formalin underwent routine anatomical dissection, during which notable vascular variations of the right TA were identified. Both specimens exhibited duplication of the right TA, and these variations were carefully documented regarding their origins, courses, and relationships to adjacent structures. A summary of the anatomical context and specific findings for each case is provided below.

Case 1

**Figure 1:**
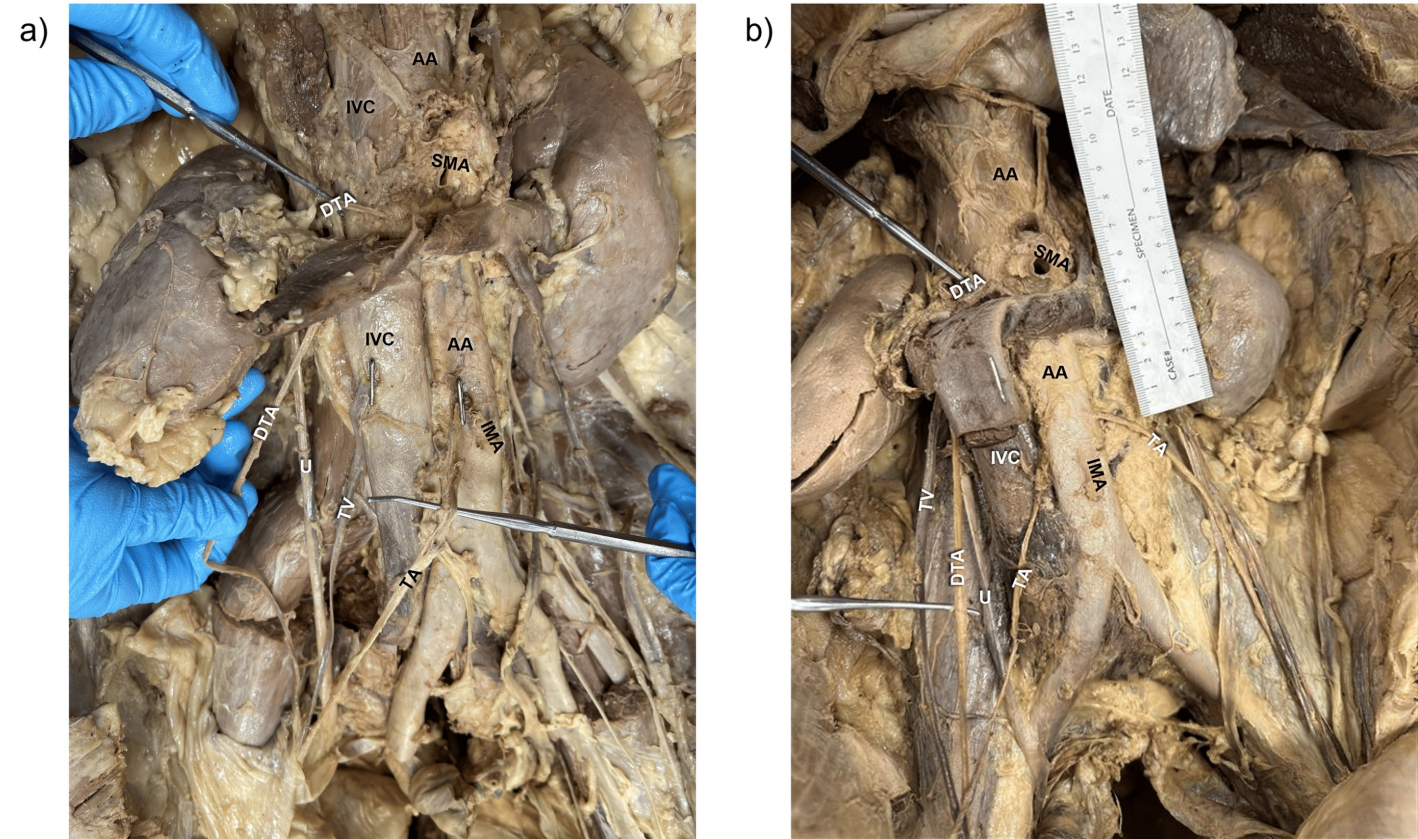
Duplicated testicular artery (DTA) identified in a 63-year-old (a) and 55-year-old (b) male cadaver. In both cadavers, the right testicular artery (TA) originates in its usual location off the abdominal aorta (AA) superior to the inferior mesenteric artery (IMA). Both DTAs arise from the AA lateral and superior to the superior mesenteric artery (SMA) and right renal artery (not shown), respectively. The DTAs traverse anterolaterally to the right ureter (U) and enter the ipsilateral deep inguinal ring, contributing to the spermatic cord.

In a 63-year-old male cadaver who died of complications from metastatic non-small-cell lung carcinoma, duplication of the right testicular artery (TA) was observed (Figure 1, panel a). The principal artery arose from the abdominal aorta just inferior to the origin of the right renal artery, consistent with the typical pattern. A second artery originated superior to the right renal artery at the L1 vertebral body, coursing posterior to it before descending anterolaterally over the right psoas major muscle and ureter. Both arteries entered the ipsilateral deep inguinal ring and contributed to the spermatic cord.

Case 2

In a 55-year-old male cadaver with no recorded cause of death, a similar duplication of the right TA was identified (Figure 1, panel b). One artery arose from the abdominal aorta inferior to the right renal artery, while the accessory vessel originated lateral to the superior mesenteric artery at the L1 vertebral level. The latter passed posterior to the right renal artery before descending independently toward the deep inguinal ring and spermatic cord. In both specimens, the left testicular artery arose as a single branch from the abdominal aorta. The kidneys, ureters, and gonads demonstrated no gross morphological abnormalities.

## Discussion

Anatomical and embryological considerations

The testicular artery originates embryologically from the lateral mesonephric branches of the dorsal aorta, which initially supply the mesonephros, suprarenal glands, and gonads. During development, multiple mesonephric arteries are present, but most regress as the definitive renal and gonadal vessels are established. Persistence of more than one artery destined for the gonad results in duplication or, more rarely, multiple testicular arteries [2,4]. The variations observed in the present study can be explained by the persistence of two separate mesonephric channels on the right side.

Prevalence of testicular artery duplication

Anatomical variations of the gonadal arteries have been extensively documented through cadaveric investigations and radiological evaluations. The incidence of duplicated testicular arteries is reported to vary greatly [1,3]. It is observed that right-sided duplications are more prevalent, whereas bilateral duplications are considered to be rare [3]. These variations are frequently identified incidentally during anatomical dissections [3], radiological assessments such as multidetector computed tomography (MDCT) angiography [1], or intraoperatively in retroperitoneal and urological procedures [5].

Paraskevas et al. detailed cases of bilateral double testicular arteries, highlighting both the rarity of this anomaly and the potential embryological mechanisms underlying such a phenomenon [3]. More recently, Konstantinos et al. documented a case in which a right testicular artery of renal origin coexisted with a duplicated renal artery, further emphasizing the close embryological relationship between the gonadal and renal vasculature [4].

Clinical implications

Although duplication of the testicular artery is generally asymptomatic and often discovered incidentally, its recognition holds significant clinical relevance. During urological procedures such as orchiectomy, varicocelectomy, and orchiopexy, inadvertent injury or ligation of an accessory artery may jeopardize testicular perfusion, potentially resulting in ischemia, impaired spermatogenesis, or recurrence of varicocele [2,3]. Similarly, in renal transplantation or retroperitoneal surgery, accessory gonadal arteries can complicate hilar dissection and elevate the risk of vascular injury if not properly identified and preserved [1,4]. From a radiological standpoint, duplication may be mistaken for aberrant vasculature or may be completely overlooked during angiographic evaluation, leading to incomplete embolization in the treatment of conditions such as varicocele or trauma-related bleeding [1,5]. Preoperative MDCT angiography has thus become an essential tool for detecting vascular anomalies, enabling surgeons and interventional radiologists to anticipate anatomical variations and mitigate intraoperative complications [1]. Ultimately, awareness of testicular artery duplication enhances surgical safety, improves treatment outcomes, and emphasizes the ongoing importance of anatomical study in guiding clinical practice.

Diagnostic approaches

MDCT angiography has emerged as the definitive modality for the detection and classification of gonadal arterial variations during preoperative vascular assessment. Balci et al. introduced a classification system founded on angiographic findings, which holds significant clinical utility for surgeons and interventional radiologists [1]. In clinical practice, such imaging modalities can mitigate intraoperative surprises and diminish the risk of vascular injury.

Significance of the present cases

The two cadaveric cases presented herein contribute to the limited body of literature concerning right-sided TA duplication. Notably, both cases involved arteries that originated independently from the abdominal aorta, with no associated renal or ureteral anomalies. These findings highlight the inherent variability of gonadal vascular anatomy and underscore the importance of meticulous attention during dissection, imaging interpretation, and surgical procedures.

## Conclusions

Duplicated TAs represent a rare anatomical variation that is most frequently identified incidentally during dissection, imaging, or surgical procedures. Although generally asymptomatic, their presence holds significant implications in the fields of urology, renal surgery, and interventional radiology. Injury or unintended ligation of an accessory artery may impair testicular perfusion and spermatogenesis, while unrecognized duplication can pose challenges during embolization or renal hilar dissection. The two cadaveric cases described herein underscore both the variability of gonadal vasculature and the significance of anatomical awareness in clinical practice. Surgeons and radiologists should maintain vigilance for these anomalies, with preoperative MDCT angiography serving as an invaluable tool in selected patients. Routine documentation of such variations during cadaveric studies contributes to safer surgical planning and augments anatomical education.

## References

[1] Balci S, Ardali Duzgun S, Arslan S, Balci H, Karcaaltincaba M, Karaosmanoglu AD (2021). Anatomy of testicular artery: a proposal for a classification with MDCT angiography. Eur J Radiol.

[2] Paraskevas GK, Ioannidis O, Raikos A, Papaziogas B, Natsis K, Spyridakis I, Kitsoulis P (2011). High origin of a testicular artery: a case report and review of the literature. J Med Case Rep.

[3] Paraskevas GK, Natsis K, Nitsa Z, Papaziogas B, Kitsoulis P (2014). Bilateral double testicular arteries: a case report and review of the literature. Potential embryological and surgical considerations. Folia Morphol (Warsz).

[4] Konstantinos N, Vasilios E, Trifon T, George T, George T, Vasileios P, Maria P (2024). A right-sided testicular artery of renal origin, in coexistence with a renal artery duplication: dissection findings with clinical significance. F1000Res.

[5] Deucher PW, Thorkildsen TN, Farrell D, Khan AA, Cornelio VC, Abouzaid KA, Imam A (2024). A cadaveric case report of an incomplete double ureter associated with testicular arterial variations. Cureus.

[6] Soni S, Wadhwa A (2010). Multiple variations in the paired arteries of abdominal aorta - clinical implications. J Clin Diagn Res.

